# A hub gene signature as a therapeutic target and biomarker for sepsis and geriatric sepsis-induced ARDS concomitant with COVID-19 infection

**DOI:** 10.3389/fimmu.2023.1257834

**Published:** 2023-09-26

**Authors:** Guojun Qian, Hongwei Fang, Anning Chen, Zhun Sun, Meiying Huang, Mengyuan Luo, Erdeng Cheng, Shengyi Zhang, Xiaokai Wang, Hao Fang

**Affiliations:** ^1^Department of Anesthesiology, Zhongshan Hospital, Fudan University, Shanghai, China; ^2^Affiliated Cancer Hospital and Institute of Guangzhou Medical University, Guangzhou, China; ^3^Department of Anesthesiology, Minhang Branch, Zhongshan Hospital, Fudan University, Shanghai, China; ^4^Department of Thoracic Surgery, Songjiang Hospital Affiliated to Shanghai Jiaotong University School of Medicine, Shanghai, China; ^5^Department of Interventional and Vascular Surgery, Xuzhou First People's Hospital, Xuzhou, China; ^6^Fudan Zhangjiang Institute, Shanghai, China; ^7^Department of Anesthesiology, Shanghai Geriatric Medical Center, Shanghai, China

**Keywords:** COVID-19, sepsis, sepsis-ARDS, hub gene, disease biomarker, bioinformatics

## Abstract

**Background:**

COVID-19 and sepsis represent formidable public health challenges, characterized by incompletely elucidated molecular mechanisms. Elucidating the interplay between COVID-19 and sepsis, particularly in geriatric patients suffering from sepsis-induced acute respiratory distress syndrome (ARDS), is of paramount importance for identifying potential therapeutic interventions to mitigate hospitalization and mortality risks.

**Methods:**

We employed bioinformatics and systems biology approaches to identify hub genes, shared pathways, molecular biomarkers, and candidate therapeutics for managing sepsis and sepsis-induced ARDS in the context of COVID-19 infection, as well as co-existing or sequentially occurring infections. We corroborated these hub genes utilizing murine sepsis-ARDS models and blood samples derived from geriatric patients afflicted by sepsis-induced ARDS.

**Results:**

Our investigation revealed 189 differentially expressed genes (DEGs) shared among COVID-19 and sepsis datasets. We constructed a protein-protein interaction network, unearthing pivotal hub genes and modules. Notably, nine hub genes displayed significant alterations and correlations with critical inflammatory mediators of pulmonary injury in murine septic lungs. Simultaneously, 12 displayed significant changes and correlations with a neutrophil-recruiting chemokine in geriatric patients with sepsis-induced ARDS. Of these, six hub genes (CD247, CD2, CD40LG, KLRB1, LCN2, RETN) showed significant alterations across COVID-19, sepsis, and geriatric sepsis-induced ARDS. Our single-cell RNA sequencing analysis of hub genes across diverse immune cell types furnished insights into disease pathogenesis. Functional analysis underscored the interconnection between sepsis/sepsis-ARDS and COVID-19, enabling us to pinpoint potential therapeutic targets, transcription factor-gene interactions, DEG-microRNA co-regulatory networks, and prospective drug and chemical compound interactions involving hub genes.

**Conclusion:**

Our investigation offers potential therapeutic targets/biomarkers, sheds light on the immune response in geriatric patients with sepsis-induced ARDS, emphasizes the association between sepsis/sepsis-ARDS and COVID-19, and proposes prospective alternative pathways for targeted therapeutic interventions.

## Introduction

Sepsis, a life-threatening condition resulting from an uncontrolled immune response to infection, can be instigated by various pathogens, including SARS-CoV-2, the virus responsible for COVID-19 (1, 2). With respiratory and gastrointestinal bacterial and viral infections being the most prevalent, sepsis accounts for nearly 20% of global deaths (3). Furthermore, severe COVID-19 presents similarities to sepsis-induced acute respiratory distress syndrome (ARDS), such as pulmonary inflammation, dense mucus secretion, microthrombosis, and systemic proinflammatory cytokine elevation (4, 5). Given the immense global impact of both conditions, it is imperative to understand the pathophysiology of sepsis/sepsis-ARDS in the context of COVID-19 and refine intensive care therapies for critically ill patients.

Geriatric patients with sepsis-induced ARDS are at a higher risk of poor outcomes when infected with COVID-19 (5–7). The interplay between COVID-19 and sepsis makes this vulnerable group particularly susceptible to respiratory failure and mortality (7, 8). Therefore, early identification of key diagnostic targets is crucial for potentially mitigating COVID-19 and sepsis-induced ARDS effects. Moreover, despite the severity of this issue, the cellular and molecular events that contribute to the effects of COVID-19 on geriatric sepsis-induced ARDS have not been clearly defined.

In this study, we employ bioinformatics and systems biology approaches to examine the effects of COVID-19 on sepsis and geriatric sepsis-induced ARDS. Our investigation aims to uncover shared cellular signaling pathways, gene networks, potential biomarkers, and therapeutic targets. Furthermore, we explore candidate drugs and their underlying molecular mechanisms for the treatment of sepsis and geriatric sepsis-ARDS patients co-infected with COVID-19. To corroborate our findings, we validate hub genes in murine sepsis-ARDS models and geriatric patients with sepsis-induced ARDS, utilizing single-cell RNA sequencing (scRNA-seq) to assess their expression patterns across various immune cell populations. This comprehensive analysis furnishes therapeutic targets/biomarkers and imparts invaluable insights into the pathogenesis of these intricate conditions.

## Materials and methods

### Study population

In this study, patient samples were collected from critically ill individuals aged 60 years or older who were undergoing treatment in the emergency intensive care units (ICUs) at Zhongshan Hospital and Minhang Hospital, both affiliated with Fudan University, China. We prospectively enrolled 17 patients diagnosed with sepsis-induced ARDS, following the diagnostic criteria established in the 2016 international Sepsis 3.0 consensus proposed by sepsis experts and the Berlin definition and diagnostic criteria for ARDS (9, 10). These patients did not have SARS-CoV-2 infection. **Table S1** presents an overview of the demographic and clinical features of the study population. For three of these patients, blood samples were collected on Day 1 and Day 7 and subsequently subjected to scRNA-seq. Based on their clinical manifestations, Day 1 and Day 7 were designated as the onset and recovery phases, respectively. For additional information, please refer to **Table S2**.

### Gene expression datasets

To investigate the shared genetic correlations between COVID-19 and sepsis, we utilized RNA-seq and microarray datasets from the Gene Expression Omnibus (GEO) database. Specifically, we analyzed the GSE171110 dataset, which comprises whole-blood RNA-seq profiles from COVID-19 patients and healthy donors, and the GSE137342 dataset, which includes whole blood cells from sepsis patients and healthy volunteers and was sequenced using microarrays (11). Further information regarding the datasets is outlined in **Table S3**.

### Identification of differentially expressed genes

DEGs were determined from expression values utilizing the “limma” package in R software (version 4.2.0), applying Benjamini-Hochberg correction to regulate the false discovery rate. The DEGs were considered significant if they met the cutoff criteria (adjusted *P-value* < 0.05 and |logFC| ≥ 1.0). The common DEGs between COVID-19 and sepsis were identified using an online VENN analysis tool called Jvenn (12).

### Gene ontology and pathway enrichment analysis

Utilizing the “clusterProfiler” package in R, potential functions and pathways associated with DEGs were identified. GO and Kyoto Encyclopedia of Genes and Genomes (KEGG) pathway analyses were performed, and a standardized metric (*P*-value < 0.05, *Q*-value < 0.25) was used to prioritize the top functional items and pathways.

### Protein-protein interaction network analysis

The PPI network was generated based on the proteins encoded by the common DEGs between COVID-19 and sepsis, as determined through the STRING database (13). The PPI network was further processed and analyzed using Cytoscape software, and gene clusters were identified using the Markov cluster method. The prominent nodes in the PPI network modules were predicted using the CytoHubba plugin to identify hub genes.

### Gene regulatory networks analysis

Hub gene-microRNA (Hub-miRNA) interaction networks and hub gene-transcription factor (Hub-TF) interaction networks were analyzed using the NetworkAnalyst tool (14). TarBase (15) and miRTarBase (16) databases were used to identify Hub-miRNA interactions, while the JASPAR database (17) was used to analyze Hub-TF interactions. The hub genes common to COVID-19 and sepsis were used in the gene regulatory networks analysis to identify the transcriptional elements and miRNA that regulate hub genes at the post-transcriptional level.

### Immune infiltration analysis

The composition of immune cells in blood samples was analyzed utilizing the CIBERSORT (18), a deconvolution algorithm designed to quantify the representation of 22 distinct subpopulations of infiltrating lymphocytes. GraphPad Prism 8.0.2 software was utilized to contrast immune cell proportions between the COVID-19 and healthy control samples, as well as between the sepsis and healthy control samples.

### Evaluation of applicant drugs

The Enrichr web server (19) and the DSigDB database (20) were used to identify pharmacological compounds connected to the common hub genes of COVID-19 and sepsis, based on a statistical threshold of adjusted *P-value* < 0.05.

### Gene-disease association analysis

The DisGeNET database (21) was utilized to investigate gene-disease associations and identify diseases and chronic issues linked to the common hub genes of COVID-19 and sepsis. The NetworkAnalyst tool (22) was also used in this analysis.

### Blood sample collection, processing, and analysis

Peripheral blood samples were collected from each patient in a 5 mL EDTA tube on the day of enrollment and processed within two hours to isolate all peripheral blood mononuclear cells (PBMCs). The PBMCs were either subjected to scRNA-seq or cryopreserved and stored at -80°C for subsequent analysis.

### scRNA-seq

Blood-derived PBMCs were subjected to single-cell gel bead-in-emulsion generation using the Chromium Controller Instrument (10×Genomics) and the Single Cell 3’ Library and Gel Bead Kit V3.1 (10×Genomics) according to the manufacturer’s recommended protocol. The resulting GEM libraries were then sequenced on an Illumina Novaseq 6000 using a custom paired-end sequencing mode of 150 bp for the first read and 150 bp for the second read. The raw data was processed using the Cell Ranger Single-Cell Software Suite (v5.0.0) with default parameters and aligned to the genomic reference. To maintain data quality, cells were discarded if they had gene counts below 200 or over 5000, or mitochondrial gene expression greater than 15%. The most variable genes among the single cells were identified, and their log-transformed gene-barcode matrices were subjected to principal component analysis to reduce their dimensionality. The resulting data was visualized in a t-SNE plot constructed with Seurat (v3.1.1) to differentiate among the PBMC cell types.

### Mouse models of sepsis-induced ARDS

To induce sepsis-induced ARDS in mice, we used the cecal ligation and puncture (CLP) method (23, 24). In brief, mice underwent an 8-hour fast and 4-hour water deprivation before surgery. Under 2% isoflurane anesthesia, a sterile abdominal incision provided access to the cecum. The cecum was ligated 1 cm from its end, punctured twice with a 22-gauge needle, repositioned, and the incision closed in two layers. Control mice experienced a similar procedure without CLP. At 24 hours post-CLP, lungs were collected, and RNA was isolated from the tissue using a Tiangen kit. RNA was reverse transcribed with the Tiangen kit, and relative gene expression was determined via the ΔΔCt method, using actin as an internal control.

### Statistical analysis

Data are displayed as mean ± standard error of the mean (SEM). Statistical analyses were executed using GraphPad Prism and R software. For normally distributed data, a two-tailed, unpaired Student’s t-test was applied to assess differences between groups. Non-normally distributed data were evaluated using the Mann-Whitney test. Correlation analyses were performed employing the Pearson method. Statistical significance was established for P-values below 0.05 (*P < 0.05; **P < 0.01; ***P < 0.001; ****P < 0.0001).

## Results

### Investigation of the genetic overlap between COVID-19 and sepsis

**Figure 1:**
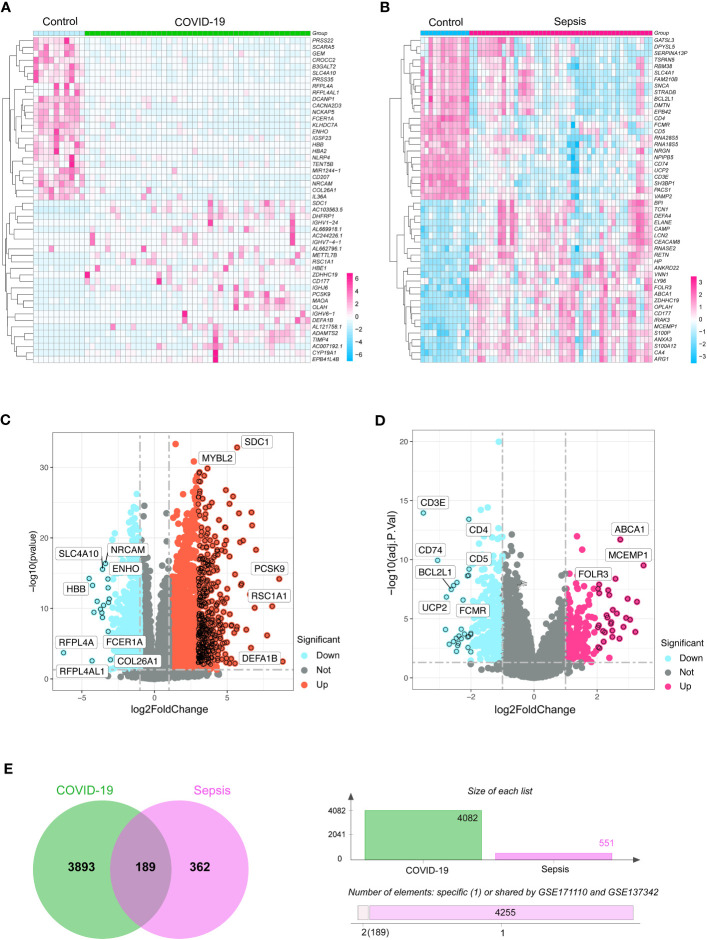
The heatmaps exhibit differentially expressed genes (DGEs) for **(A)** COVID-19 (GSE171110) and **(B)** sepsis (GSE137342) cases. Volcano diagrams represent the DGEs for **(C)** COVID-19 and **(D)** sepsis. The DEGs were considered significant if they met the cutoff criteria (adjusted *P-value* < 0.05 and |logFC| ≥ 1.0). **(E)** A Venn chart highlights the overlapping DGEs in both COVID-19 and sepsis situations.

To reveal shared genetic pathways between COVID-19 and sepsis, we comprehensively analyzed human transcriptomic data from the GEO database (**Figure S1**). Our results showed that COVID-19 and sepsis patients had 4082 and 551 DEGs, respectively, compared to healthy controls (**Table S3**). The DEGs of the highest significance for COVID-19 and sepsis are depicted in heatmaps and volcano plots in **Figures 1A–D**. We then utilized Jvenn to uncover 189 DEGs that were shared between the sepsis and COVID-19 datasets (**Figure 1E**). The complete list of these 189 DEGs can be found in **Table S4**.

### Functional enrichment analysis of GO terms and significant signaling pathways

**Figure 2:**
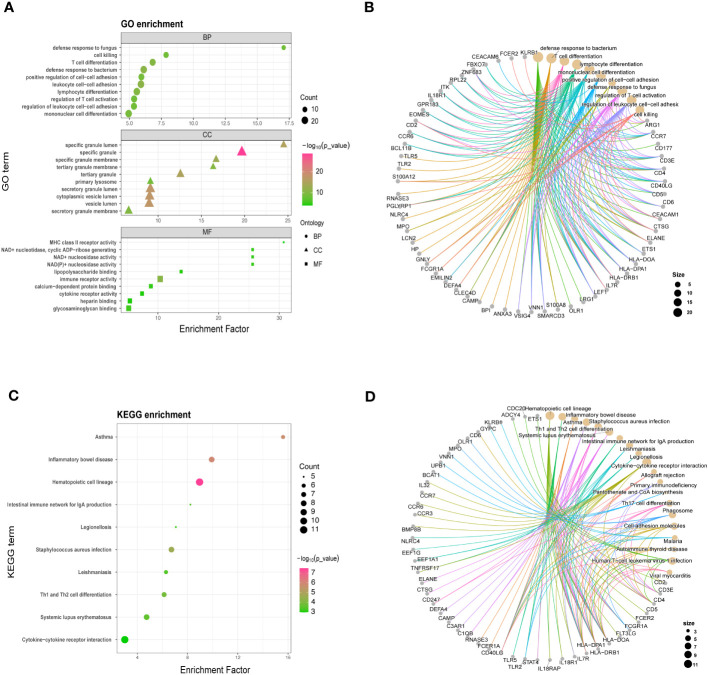
GO **(A, B)** and KEGG analysis **(C, D)** for shared differentially expressed genes in COVID-19 and sepsis. GO and KEGG pathway analyses were conducted, prioritizing significant functional items and pathways based on a standardized metric (*P* < 0.05, *Q* < 0.25).

To understand the functional significance of the shared DEGs between COVID-19 and sepsis, we conducted a functional enrichment analysis using the “clusterProfiler” package. The analysis consisted of GO enrichment, linking genes to GO terms, and KEGG pathway enrichment, which revealed gene-pathway associations. The GO analysis was categorized into biological process, cellular component, and molecular function (25). Our results showed that the DEGs were significantly enriched in defense against fungus in the biological process category, specific granule lumen in the cellular component category, and MHC class II receptor activity in the molecular function category (**Figures 2A, B**). The KEGG pathway analysis revealed the top 10 pathways, including asthma, inflammatory bowel disease, hematopoietic cell lineage, intestinal immune network for IgA production, legionellosis, staphylococcus aureus infection, leishmaniasis, Th1 and Th2 cell differentiation, systemic lupus erythematosus, and cytokine-cytokine receptor interaction (**Figures 2C, D**).

### Functional networks and hub genes identified by PPI analysis

**Figure 3:**
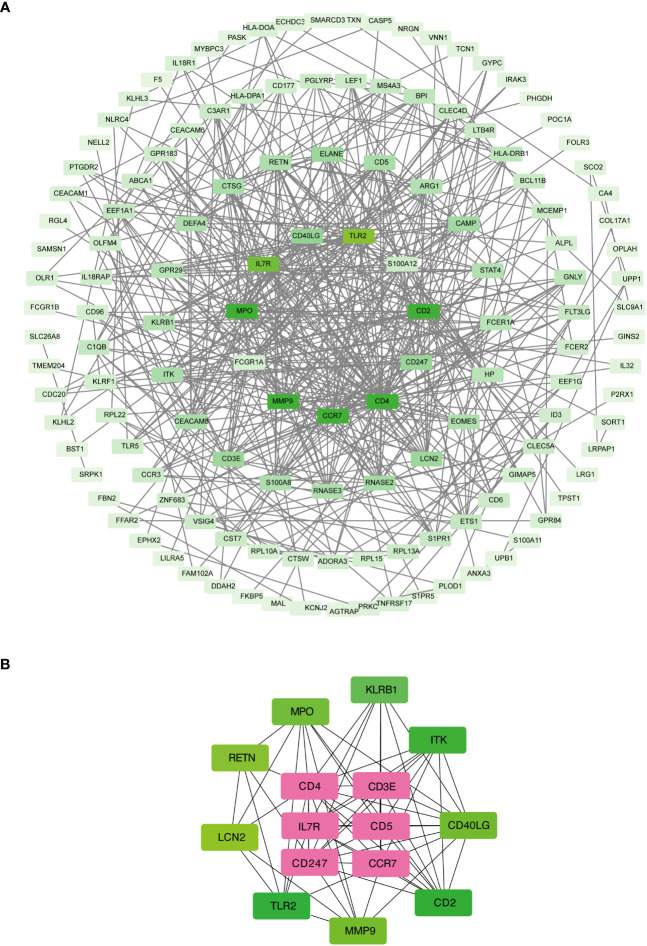
Protein-protein interaction network **(A)** and hub genes **(B)** of shared differentially expressed genes in both COVID-19 and sepsis cases. This network underwent processing and analysis in Cytoscape. Prominent nodes within the network modules were predicted using the CytoHubba plugin to identify hub genes.

The shared DEGs between COVID-19 and sepsis were subjected to a PPI analysis using the STRING database, aiming to reveal functional networks and associated pathways. The PPI network is illustrated in **Figure 3A**, where highly interconnected nodes represent hub genes. The maximal clique centrality method of cytoHubba in Cytoscape was employed to identify the top 15 most influential DEGs, which include CD4, CD3E, IL7R, CD5, CD247, CD2, CCR7, CD40LG, ITK, KLRB1, MPO, MMP9, TLR2, LCN2, and RETN. These hub DEGs hold potential as biomarkers and therapeutic targets for COVID-19 and sepsis, presenting novel opportunities for therapeutic intervention. A submodule network was constructed to facilitate a better understanding of the relationships and positions of these genes, as shown in **Figure 3B**.

### Validation of the identified hub genes

**Figure 4:**
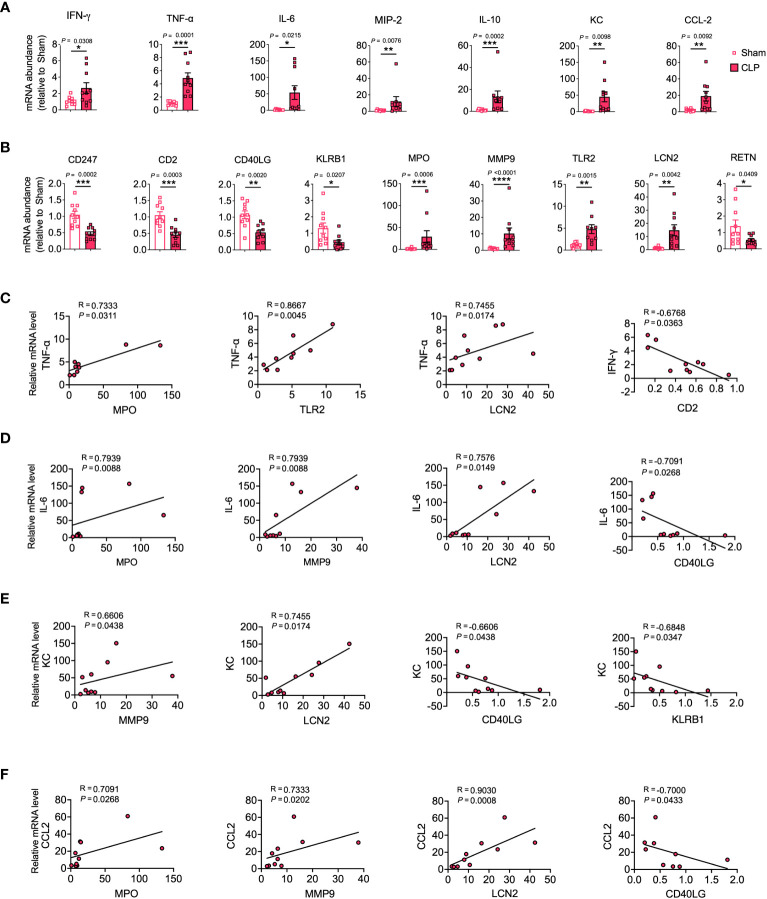
Validation of hub genes through a mouse model of sepsis-induced ARDS. mRNA expression levels of cytokines, chemokines **(A)**, and hub genes **(B)** were assessed in lung tissue homogenates. **(C–F)** Relevant scatterplots were created to study the relationships between mRNA expression levels of hub genes and TNF-α, IFN-γ, IL-6, KC, and CCL2 in the lungs using Spearman’s rank correlation. **(A–F)** Data from two experiments were combined, with a total of *n* = 10 mice. **(A, B)** Student’s *t*-test.

To assess the potential of hub genes as biomarkers for predicting COVID-19 and sepsis-induced ARDS, we employed a murine model of sepsis-induced ARDS as a proxy for both COVID-19 and sepsis-related lung injuries. This choice was guided by the shared pathophysiological characteristics of ARDS in COVID-19 and sepsis, and their substantial mechanistic overlap. The use of the murine model was also influenced by the absence of a biosafety level 3 facility in our laboratory, which precluded the development of a specific SARS-CoV-2 infection model. Our results showed that genes such as IFN-γ, TNF-α, IL-6, MIP-2, IL-10, KC (CXCL1), CCL-2, MPO, MMP9, TLR2, and LCN2 were significantly upregulated in the lungs of sepsis-induced ARDS mice compared to control mice. In contrast, genes such as CD247, CD2, CD40LG, KLRB1, and RETN were significantly downregulated (**Figures 4A, B**).

TNF-α, IFN-γ, and IL-6 represent vital inflammatory mediators in the progression of lung injury in both sepsis and COVID-19 scenarios (1, 26). Positive correlations were observed between MPO, TLR2, LCN2 and TNF-α, and between MPO, MMP9, LCN2 and IL-6. Conversely, negative correlations were observed between CD2 and IFN-γ, and between CD40LG and IL-6 (**Figures 4C, D**, **S2A–C**).

KC and CCL-2 serve as significant chemokines that attract neutrophils and monocytes to infection sites, thereby playing a pivotal role in the pathophysiology of COVID-19 and sepsis-induced lung injury (1, 27, 28). The results showed that MMP9 and LCN2 were positively correlated with KC, while CD40LG and KLRB1 were negatively correlated with KC. Furthermore, MPO, MMP9, and LCN2 were positively correlated with CCL-2, while CD40LG was negatively correlated with CCL-2 (**Figures 4E, F**, **S2D, E**).

**Figure 5:**
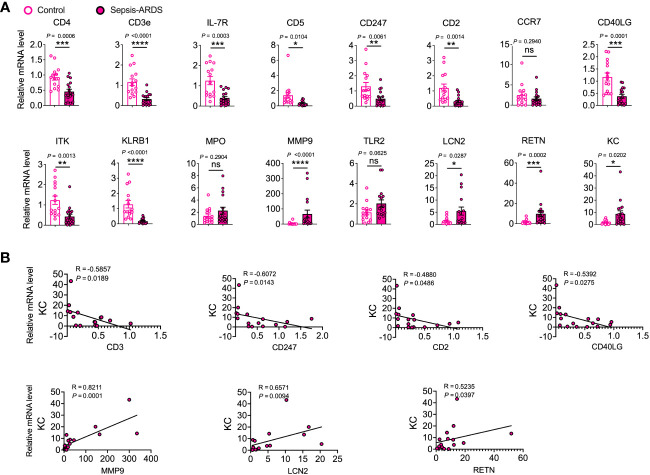
Validation of hub genes in elderly sepsis-induced ARDS. **(A)** mRNA expression levels of the identified hub genes and chemokine (KC) were evaluated in PBMCs. *n* = 15–17. Student’s *t*-test and Mann-Whitney test. **(B)** Appropriate scatterplots were generated to investigate the association between mRNA expression levels of hub genes and KC in PBMCs utilizing Spearman’s rank correlation coefficient (R).

Finally, we assessed the potential of the discovered hub genes as targeted biomarkers in elderly sepsis-induced ARDS. We investigated the expression levels of these hub genes in PBMCs collected from elderly patients with sepsis-induced ARDS. Our findings revealed significant decreases in CD4, CD3e, IL-7R, CD5, CD247, CD2, CD40LG, ITK, and KLRB1, and significant elevations in MMP9, LCN2, and RETN in sepsis-induced ARDS compared to controls (**Figure 5A**). Similar results were also observed in COVID-19 and sepsis (**Figure S3**). Moreover, we found that CD3, CD247, CD2, and CD40LG were negatively correlated with KC, whereas MMP-9, LCN2, and RETN were positively correlated with KC (**Figure 5B**), suggesting that these candidate hub genes may regulate geriatric sepsis-induced ARDS by modulating the recruitment of neutrophils to the lung.

### scRNA-seq of PBMCs from elderly patients with sepsis-induced ARDS

**Figure 6:**
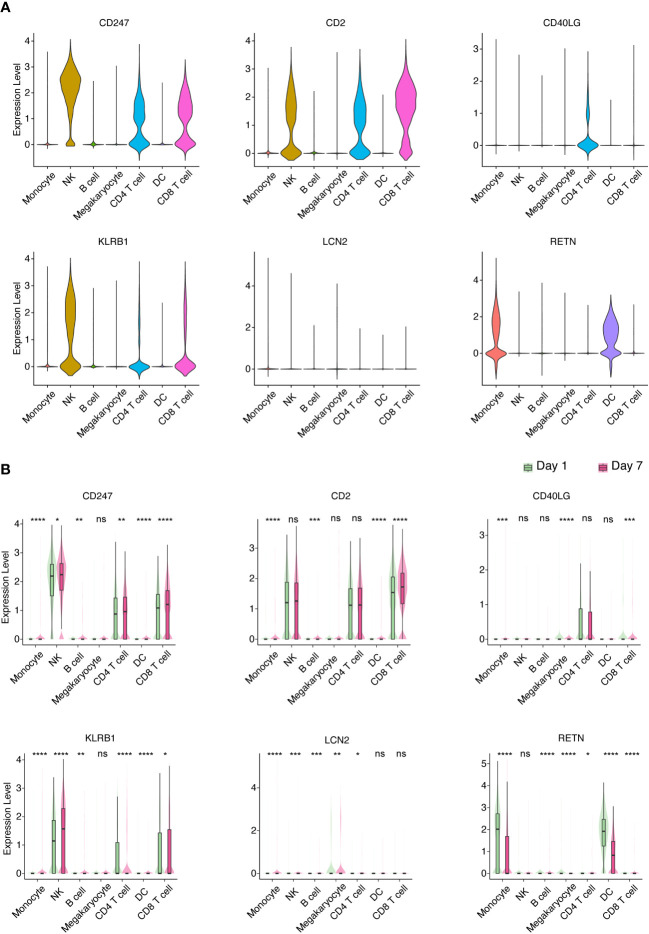
Identification of key PBMC populations expressing hub genes in aged sepsis-induced ARDS. **(A)** Detection of the primary PBMC cell population expressing hub genes in patients with elderly ARDS using scRNA-seq. **(B)** Differential expression of selected hub genes in various PBMC cell populations throughout disease progression. **(A, B)** Student’s t-test and Mann-Whitney test.

In order to provide additional support for the involvement of hub genes in the development of sepsis-induced ARDS among elderly individuals, we executed scRNA-seq on PBMCs procured from geriatric patients diagnosed with sepsis-induced ARDS. Drawing upon the insights gleaned from our murine sepsis-induced ARDS model and human subjects afflicted with sepsis-induced ARDS, we selected CD247, CD2, CD40LG, KLRB1, LCN2, and RETN as the foci of our subsequent investigation. Our findings revealed that CD247, CD2, and KLRB1 were predominantly expressed in natural killer (NK) cells, CD4+ T cells, and CD8+ T cells, respectively; CD40LG was chiefly expressed in CD4+ T cells; LCN2 was primarily expressed in monocytes; and RETN was principally expressed in monocytes and DCs (**Figures 6A**, **S4**). Additionally, we detected elevated expression of CD247 in NK cells, CD4+ T cells, and CD8+ T cells on Day 7 (recovery phase) in comparison to Day 1 (onset phase). CD2 expression was heightened in CD8+ T cells on Day 7 relative to Day 1. CD40LG expression demonstrated a decline in CD8+ T cells on Day 7 compared to Day 1. KLRB1 expression exhibited an increase in NK cells and CD8+ T cells and a decrease in CD4+ T cells on Day 7 relative to Day 1. LCN2 expression witnessed an augmentation in monocytes on Day 7 in comparison to Day 1. Lastly, RETN expression manifested a reduction in monocytes and DCs on Day 7 as opposed to Day 1 (**Figure 6B**). These findings indicate that the six identified hub genes could be closely related to the development of sepsis-induced ARDS in older individuals, with their expression patterns and alterations potentially having a crucial impact on disease progression and recovery.

### Immune infiltration analysis

**Figure 7:**
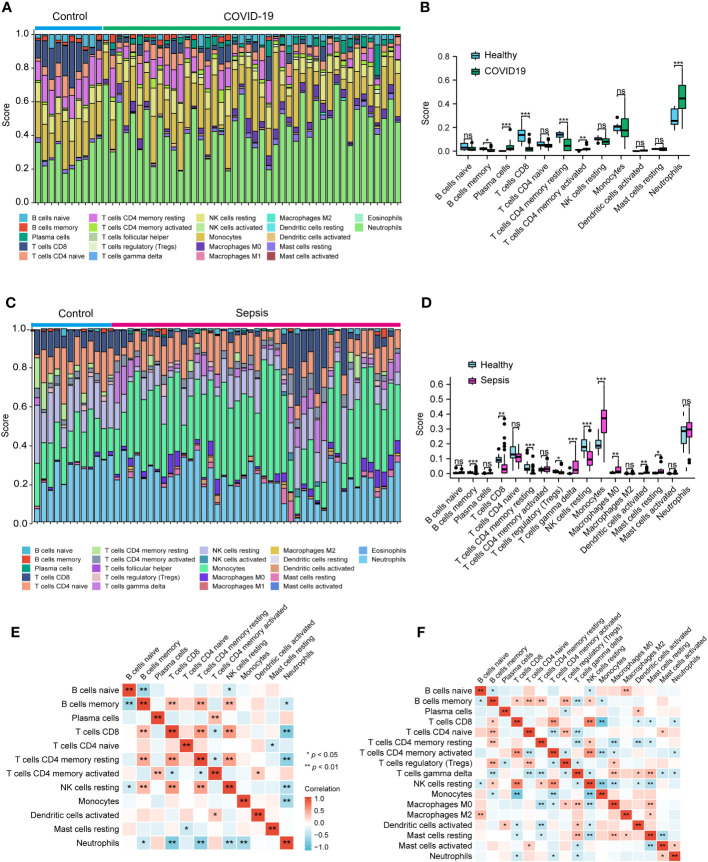
Analysis of the immune infiltration levels in the COVID-19 and sepsis. The ratio of immune cells in COVID-19 **(A, B)** and sepsis **(C, D)** were analyzed using CIBERSORT. The relationship between each immune cell for COVID-19 **(E)** and Sepsis **(F)**. **(B, D)** Student’s t-test. **(E, F)** Spearman.

To delve deeper into the potential roles of the discovered hub genes in developing COVID-19 and sepsis, we examined the proportions of immune cells and their associations with these hub genes in patients affected by COVID-19 and sepsis. Our results revealed that memory B cells, CD8+ T cells, and CD4+ memory resting T cells were reduced in COVID-19, while plasma cells, CD4+ memory activated T cells, and neutrophils were increased when compared to healthy controls (**Figures 7A, B**). Similarly, memory B cells, CD8+ T cells, CD4+ memory resting T cells, and resting NK cells were decreased in sepsis, while gamma delta T cells, monocytes, M0 macrophages, activated DCs, and resting mast cells were increased when compared to healthy controls (**Figures 7C, D**). Neutrophil levels were also elevated in sepsis, although not to a statistically significant extent. Furthermore, our analysis showed strong associations between multiple immune cell types, including memory B cells, CD8+ T cells, CD4+ memory resting T cells, and neutrophils, in both COVID-19 and sepsis (**Figures 7E, F**). Specifically, in COVID-19, memory B cells, CD8+ T cells, and CD4+ memory resting T cells were negatively correlated with neutrophils (**Figure 7E**). In sepsis, CD8+ T cells and gamma delta T cells were negatively correlated with neutrophils (**Figure 7F**).

**Figure 8:**
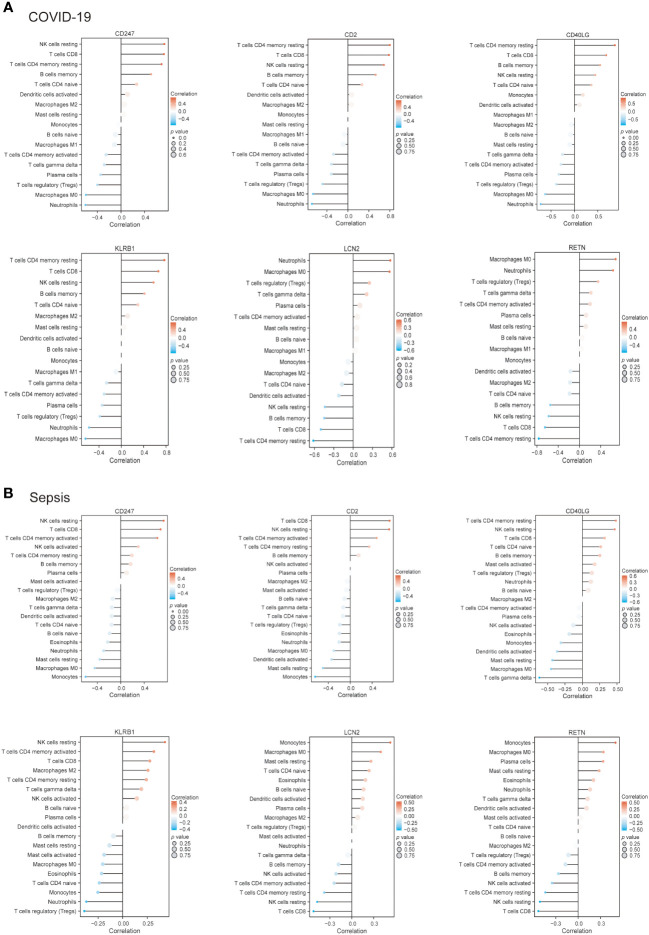
The connection between immune cells and hub genes in COVID-19 **(A)** and sepsis **(B)**. The association between the two factors was assessed employing Spearman’s rank correlation coefficient.

Subsequently, we employed the Pearson correlation coefficient to assess the association between immune cell abundance and hub gene expression in both COVID-19 and sepsis cases. Results revealed a negative correlation between neutrophils and CD247, CD2, and KLRB1, and a positive correlation with RETN in both COVID-19 and sepsis (**Figures 8A, B**). LCN2 was positively correlated with neutrophils in COVID-19 but not in sepsis cases (**Figures 8A, B**). In sepsis, monocytes were negatively correlated with CD247, CD2, and CD40LG, while positively correlated with LCN2 and RETN (**Figure 8B**). This correlation was weaker in COVID-19. CD247, CD2, CD40LG, and KLRB1 displayed a positive correlation with CD8+ T cells and CD4+ T cells in both COVID-19 and sepsis cases, while LCN2 and RETN showed a negative correlation (**Figures 8A, B**). These results demonstrate the key relationships between immune cells and hub gene expressions, highlighting differences and similarities in immune responses across COVID-19 and sepsis-induced ARDS cases.

### Network-based analysis of transcriptional and post-transcriptional regulators

**Figure 9:**
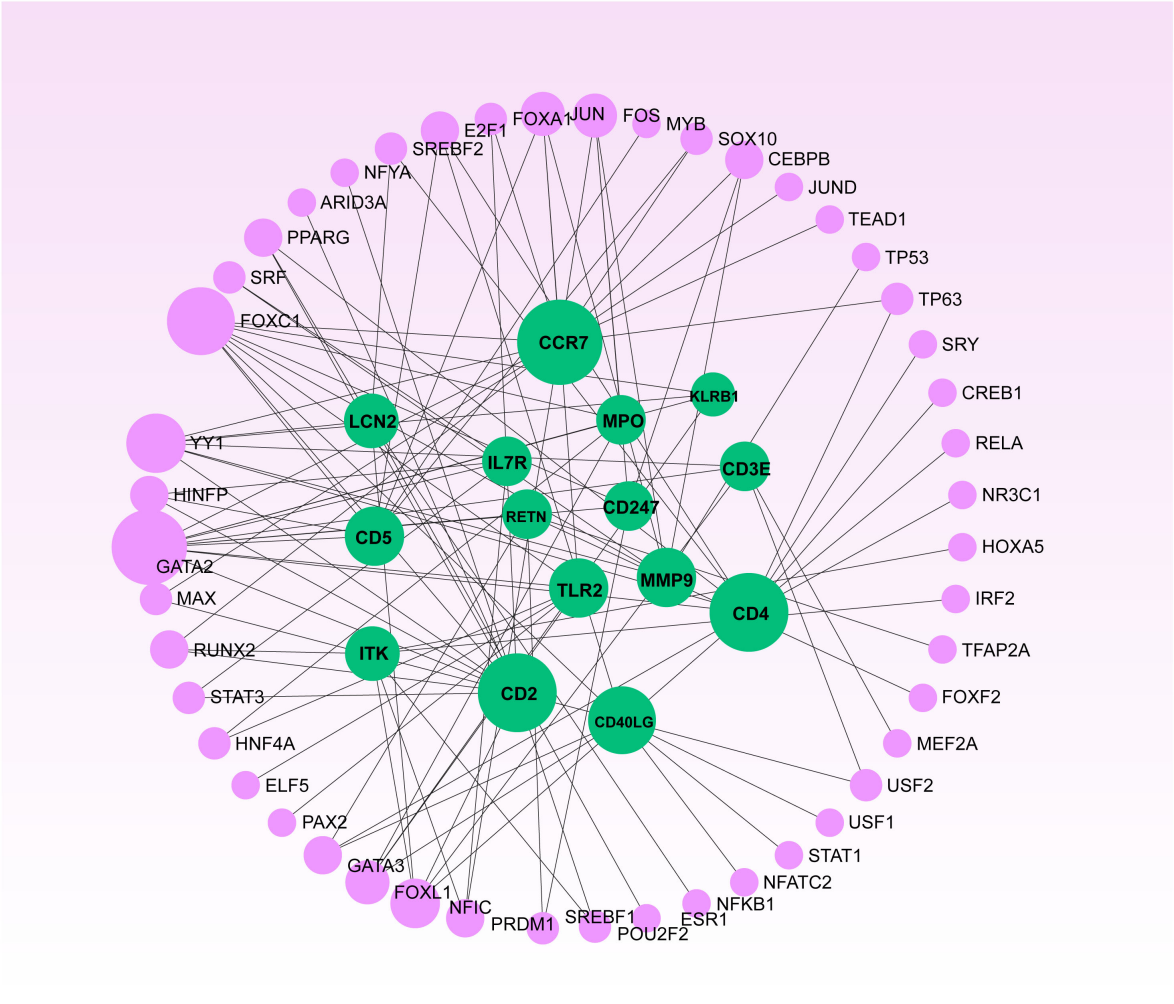
The Network Analyst produced a regulatory interaction network linking DEGs and Transcription Factors (TFs). In this network, purple nodes represent TFs, while green nodes illustrate the connections between gene symbols and TFs.

**Figure 10:**
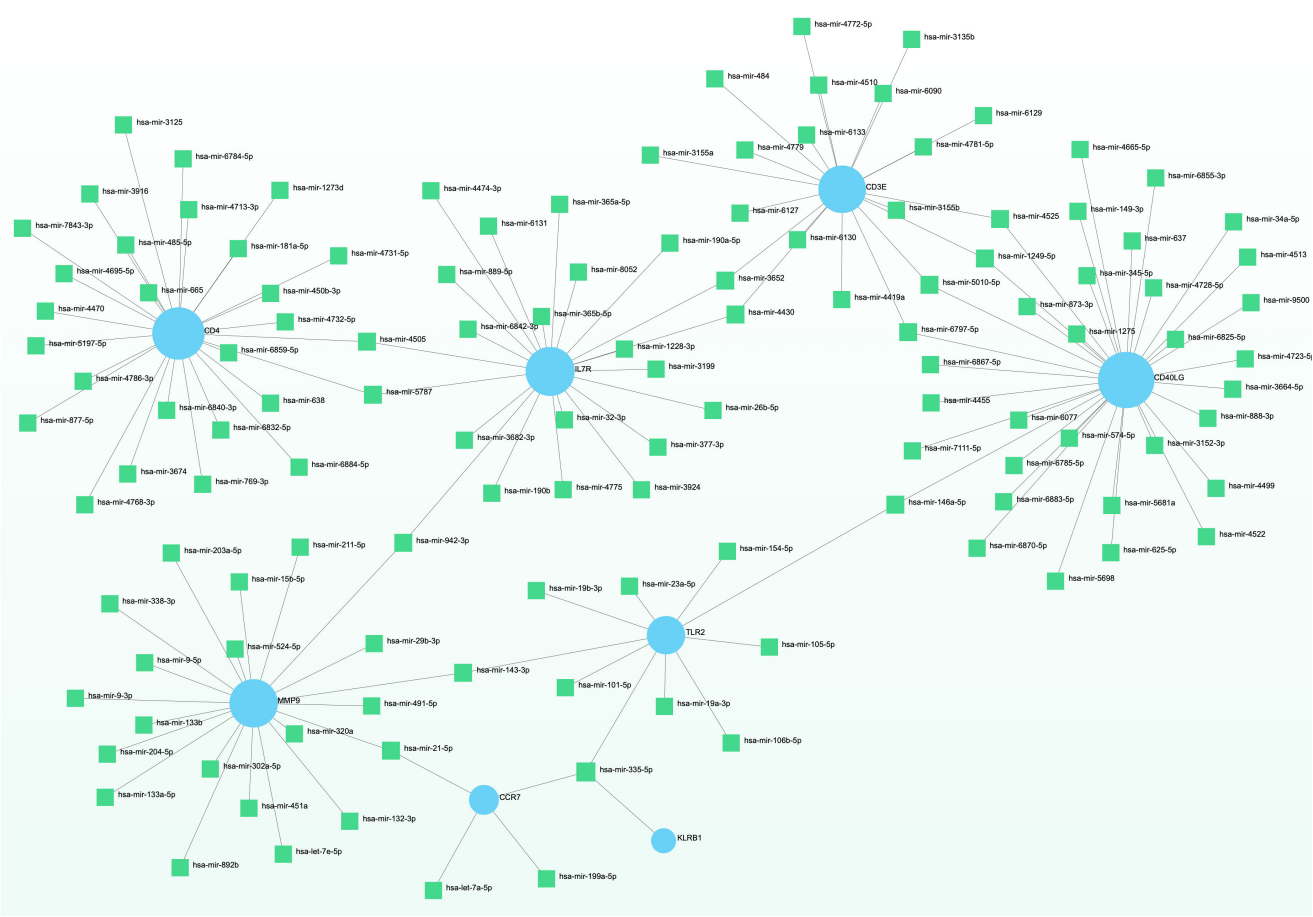
The displayed regulatory interaction network emphasizes the interconnectivity of DEGs and microRNAs. Within this network, blue circular nodes symbolize genes that interact with microRNAs.

To elucidate the regulatory molecules controlling the identified hub genes (including CD4, CD3E, IL7R, CD5, CD247, CD2, CCR7, CD40LG, ITK, KLRB1, MPO, MMP9, TLR2, LCN2, and RETN) at the transcriptional level, we utilized a network-based approach to identify the key TFs and miRNAs involved. The interaction between TF regulators and hub genes is depicted in **Figure 9**, while **Figure 10** shows the interactions of miRNA regulators with the hub genes. Our analysis revealed a total of 62 TFs and 125 miRNAs that are potentially involved in regulating the hug genes, indicating a significant interplay between them. For more detailed information, **Tables S5**, **S6** provide the regulatory network of target TF-genes and target miRNA-genes, as well as the topology table.

### Identification of potential therapeutic drug molecules

Protein-drug interaction analysis is critical for understanding the structural features of receptor sensitivity, which can aid in discovering new drugs (29). To identify potential therapeutic drugs for COVID-19, sepsis, and geriatric sepsis-ARDS, we focused on the hub genes common to these diseases. By utilizing the Enrichr tool and analyzing transcriptional characteristics from the DSigDB database, we identified ten candidate compounds. These drugs were selected based on their *P*-values and are listed in **Table 1**. Our findings suggest that these compounds may have promising therapeutic effects and could serve as the basis for developing new treatment options for these diseases.

**Table 1:** The recommended medications.

Name	*P-*value	Chemical Formula	Structure
Etynodiol	3.70E-10	C_20_H_28_O_2_	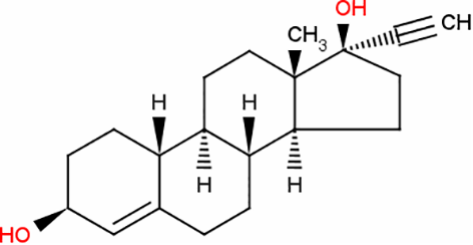
Diphenylpyraline	6.99E-08	C_19_H_23_NO	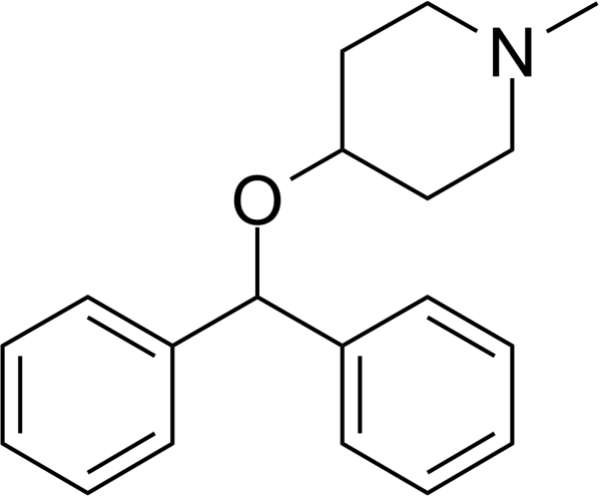
Trimethoprim	2.83E-07	C_14_H_18_N_4_O_3_	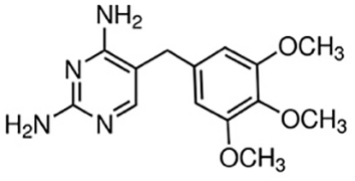
Tetradioxin	6.23E-07	C_12_H_4_Cl_4_O_2_	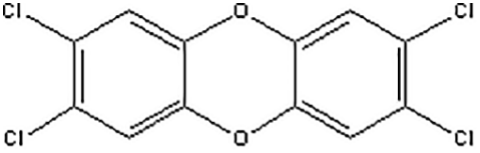
Alclometasone	7.09E-7	C_22_H_29_ClO_5_	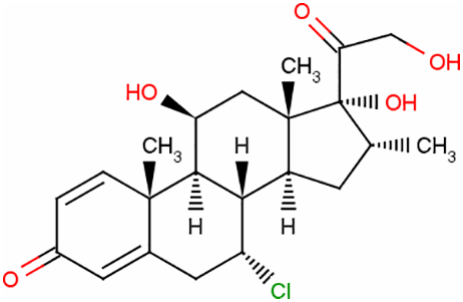
Isoflupredone	1.41E-6	C_21_H_27_FO_5_	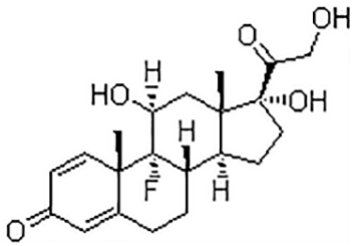
Glycoprotein	1.91E-06	C_28_H_47_N_5_O_18_	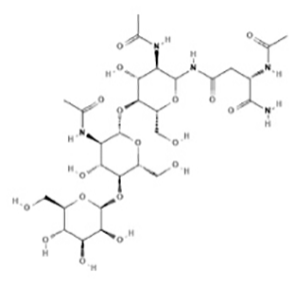
Vitamin D3	3.81E-06	C_27_H_44_O	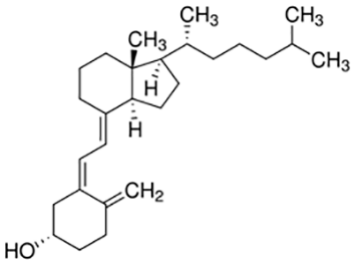
Fludroxycortide	5.88E-06	C_24_H_33_FO_6_	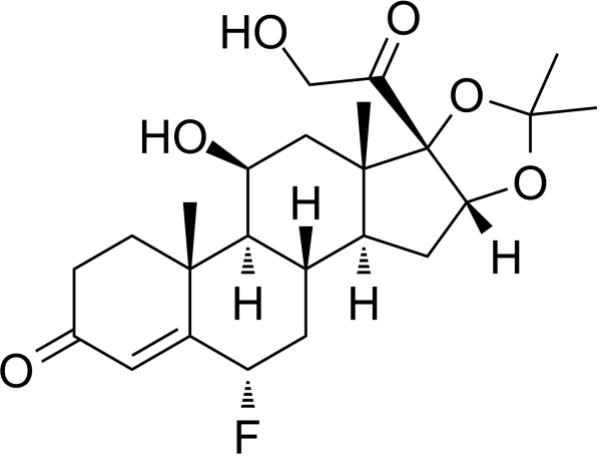

### Identification of disease association

**Figure 11:**
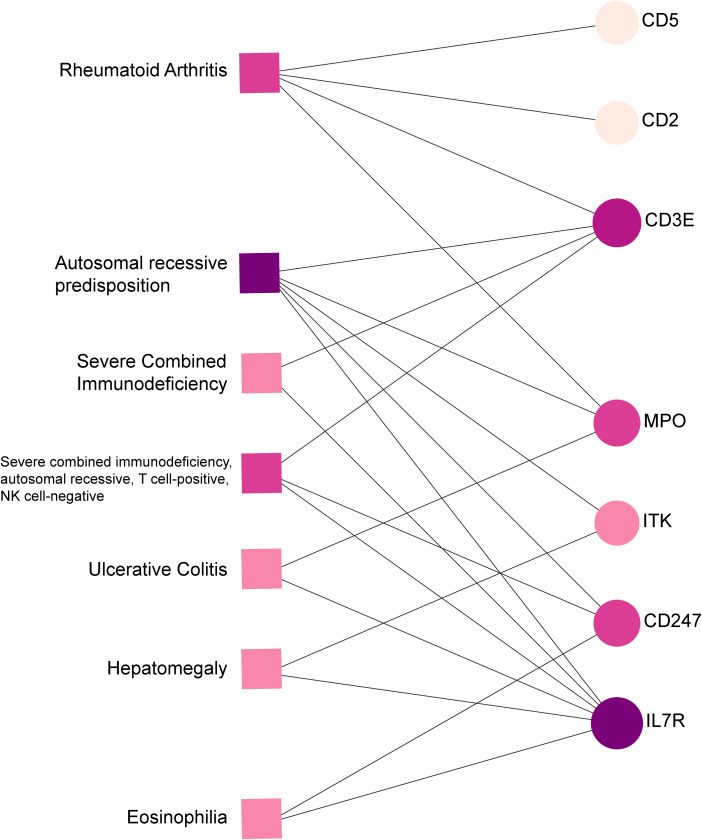
Gene-disease connection network. Square nodes symbolize diseases, and circular nodes indicate gene symbols interacting with the associated disease.

The relationship between diseases can be established based on shared genetic factors, which is a crucial step toward developing effective treatments for various disorders (30). Using NetworkAnalyst, we thoroughly examined the associations between our identified hub genes and various disease states. Our analysis revealed that autosomal recessive predisposition, rheumatoid arthritis, ulcerative colitis, severe combined immunodeficiency, hepatomegaly, eosinophilia, COVID-19, and sepsis have the strongest connections with our reported hub genes. **Figure 11** illustrates the gene-disease relationships, highlighting the potential correlations between these diseases. This emphasizes the potential impact of these diseases on each other and provides valuable insight into their complex relationships.

## Discussion

The COVID-19 pandemic and sepsis represent significant public health challenges. The close association between COVID-19 and sepsis is well-established, despite a limited understanding of the underlying molecular pathways (5, 27). Studies indicate that the severity of both COVID-19 and sepsis are mutually influenced, underscoring the need to understand the association between these conditions (2, 27, 31). Both diseases can cause severe symptoms, including ARDS, organ damage, immune system dysregulation, and long-term complications. Recent research indicates a genetic correlation between COVID-19 and sepsis, supporting that they share a common underlying biological mechanism (31, 32). Older adults are particularly vulnerable to COVID-19 and sepsis, experiencing higher rates of complications and case fatality (3, 6). Therefore, understanding the association between these conditions is crucial for improving treatment strategies, especially for geriatric patients with sepsis-induced ARDS.

Identifying common genetic pathways between COVID-19 and sepsis provides valuable insights into the underlying mechanisms of both illnesses. Our study identified 189 common DEGs in COVID-19 and sepsis, significantly enriched in defense response to fungus, specific granule lumen, MHC class II receptor activity, and various signaling pathways. Fungal infections are a risk factor for sepsis and COVID-19, and gene expression differences in sepsis are involved in the defense response to fungus (33). The lumen of specific granules, predominantly present in mature neutrophils, may hold considerable importance in modulating COVID-19 and sepsis (32), while MHC class II receptors instigate the immune response and serve as a vaccine target (34). Manipulation of MHC class II expression or signaling presents a potential therapeutic strategy for ameliorating outcomes in COVID-19 and sepsis. Further inquiry is merited to explicate the direct association between specific granule lumen/MHC class II receptor activity and these afflictions, as well as to corroborate the prospective therapeutic approaches intimated by our findings.

KEGG pathway analysis revealed several shared pathways between sepsis and COVID-19, including asthma, inflammatory bowel disease (IBD), hematopoietic cell lineage, intestinal immune network for IgA production, legionellosis, staphylococcus aureus infection, leishmaniasis, Th1 and Th2 cell differentiation, systemic lupus erythematosus, and cytokine-cytokine receptor interactions. We and others have shown a strong relationship between COVID-19 and asthma, and further connections have been established between COVID-19, asthma, and legionellosis (35, 36). IBD, COVID-19, and sepsis share a link to the gut microbiota, which is a critical factor in regulating the host’s susceptibility to SARS-CoV-2 infection and clearance (37, 38). The gut microbiota also impacts the host’s response and tolerance to treatment drugs for IBD and sepsis (38, 39). Identifying and understanding these common pathways can provide valuable insights into potential therapeutic targets and strategies for COVID-19 and sepsis management.

We have identified top hub genes (CD4, CD3E, IL7R, CD5, CD247, CD2, CCR7, CD40LG, ITK, KLRB1, MPO, MMP9, TLR2, LCN2, and RETN) associated with both sepsis and COVID-19. These hub genes may serve as important therapeutic targets or biomarkers for both diseases. In particular, the upregulation of MPO, MMP9, TLR2, and LCN2 in the lungs of sepsis-induced ARDS mice suggests their crucial role in developing lung injury in COVID-19 and sepsis. Positive correlations between MPO, TLR2, LCN2, TNF-α, and IL-6 also suggest their potential as targets for reducing inflammation and lung injury in these diseases. TLR2 has been shown to sense the SARS-CoV-2 envelope protein, leading to the production of inflammatory cytokines (40). In addition, MPO and LCN2 have been identified as critical genes in sepsis and sepsis-related ARDS, with LCN2 showing diagnostic value in sepsis-related ARDS (41).

In contrast, negative correlations observed between CD2 and IFN-γ, and between CD40LG and IL-6, as well as the downregulation of CD247, CD2, CD40LG, KLRB1, and RETN in the lungs of sepsis-induced ARDS mice, suggest their potential role in modulating the immune response and reducing inflammation in COVID-19 and sepsis. Bioinformatics and meta-analysis have identified CD247 as a critical gene for septic shock, making it a promising candidate for becoming a new biomarker for this condition (42).

Moreover, in elderly sepsis-induced ARDS patients compared to controls, we observed a significant decrease in CD4, CD3e, IL-7R, CD5, CD247, CD2, CD40LG, ITK, and KLRB1 gene expression levels, and a substantial elevation of MMP9, LCN2, and RETN, indicating their potential as targeted biomarkers for predicting COVID-19 and sepsis-induced ARDS in elderly patients. The negative correlations between CD3, CD247, CD2, CD40LG, and the positive correlations between MMP-9 and LCN2, and RETN with KC suggest their potential role in regulating neutrophil recruitment to the lung and modulating lung injury in these diseases. LCN2 and RETN are proteins produced by neutrophils and stored in their secondary granules, which are released upon neutrophil activation (43).

In the multifaceted pathogenesis of COVID-19 and sepsis-induced ARDS, the uncontrolled activation and subsequent degranulation of neutrophils are central (44, 45). These key cells in the innate immune response can, when dysregulated, release excessive granules containing proteolytic enzymes and reactive oxygen species, leading to substantial cytotoxicity (45, 46). This process damages the lung’s alveolar epithelial cells and disrupts the extracellular matrix, contributing to endothelial barrier dysfunction (46). Simultaneously, the release of inflammatory mediators like cytokines and chemokines can amplify the inflammatory response into a “cytokine storm,” potentially causing systemic inflammation that affects multiple organs (28, 44, 45, 47). Hence, our findings offer new insights into the molecular mechanisms underlying COVID-19 and sepsis-induced ARDS and suggest CD3, CD247, CD2, CD40LG MMP-9, LCN2, and RETN as the potential targets for neutrophils in developing novel therapies and diagnostic tools for these diseases.

By conducting scRNA-seq of PBMCs from elderly patients with sepsis-induced ARDS, we obtained further evidence for the role of hub genes in the pathogenesis of these conditions. The expression patterns and changes of these genes may play a crucial role in the disease’s progression and recovery. Moreover, the analysis of immune infiltration indicated changes in immune cell proportions in COVID-19 and sepsis compared to healthy controls, with strong associations found between multiple immune cell types and hub genes. These findings provide valuable insights into the relationships between immune cells and hub gene expressions, highlighting similarities and differences in immune responses across COVID-19 and sepsis-induced ARDS cases. However, further research is necessary to validate the potential of these identified hub genes as targeted biomarkers for these conditions.

At the molecular level, we observed significant connections between COVID-19 and sepsis, particularly in the context of TFs and miRNAs. Both TFs and miRNAs play crucial roles in regulating gene expression, with TFs modulating mRNA expression and miRNAs acting post-transcriptionally via RNA silencing (30, 35). The identified TFs, including GATA3, STAT1, IRF2, NFKB1, RELA, and FOXC1, and miRNAs, such as hsa-mir-335-5p, hsa-mir-4505, hsa-mir-143-3p, hsa-miR-26b-5p, and hsa-miR-146a-5p, are associated with a range of respiratory diseases, including asthma, ARDS, and pulmonary fibrosis, as well as the pathogenesis and exacerbation of sepsis and COVID-19 (32, 35, 48–50). Intriguingly, many of these miRNAs have also been implicated in various types of cancer, including lung and gastric cancer (32, 35, 51).

Our gene-disease analysis further revealed relationships between identified hub genes and various disorders, including COVID-19 and sepsis. A notable finding is the identification of several genes related to severe combined immunodeficiency (SCID). Patients with SCID may be more susceptible to COVID-19, highlighting the need for additional preventive and therapeutic measures for this vulnerable population (52). Due to limited data, the safety and efficacy of COVID-19 vaccines for SCID patients remain uncertain. Moreover, our results suggest that individuals with COVID-19 and sepsis may also be affected by other disorders such as autosomal recessive predisposition, rheumatoid arthritis, ulcerative colitis, hepatomegaly, and eosinophilia.

We have also identified potential drug molecules with therapeutic value for COVID-19, sepsis, and geriatric sepsis-induced ARDS. Among them are several compounds with immunomodulatory effects, such as Etynodiol, Glycoprotein, and Vitamin D3, which may represent promising treatment options by modulating the immune response in affected patients. Diphenylpyraline, an antihistamine drug, may offer a therapeutic effect on COVID-19 and sepsis by affecting cytokine transport and release. Corticosteroids, including Alclometasone, Isoflupredone, and Fludroxycortide, commonly used for skin inflammation (53), can potentially suppress an overactive immune system in COVID-19 or sepsis, thereby reducing inflammation and mortality risk. It is important to note, however, that not all corticosteroids are suitable for these patients, and the World Health Organization currently recommends dexamethasone or hydrocortisone for severe or critical patients with COVID-19. While Alclometasone, Isoflupredone, and Fludroxycortide may hold promise as treatments for COVID-19 and sepsis, there is insufficient evidence to support their efficacy in these conditions. Further research is necessary to determine their effectiveness and safety in treating these diseases.

While revealing preliminary insights, this study has key limitations. Our bioinformatic findings require rigorous validation through conditional knockout and pharmacological testing to move from correlation to causation and assess clinical viability. The speculative therapeutic targets need extensive experimental characterization for safety and efficacy before consideration for treatments. Additionally, our focus on expression overlooks determinants like post-translational modifications, necessitating a comprehensive systems perspective. Critically, we lacked access to facilities for SARS-CoV-2 animal models and clinical samples collection, preventing experimental validation and highlighting the need for expanded biosafety infrastructure to enable COVID-19 research. Overall, these results should be interpreted as early findings that point to future research directions rather than definitive conclusions. Our study elucidates pathways and biomarkers but requires meticulous *in vitro* and *in vivo* follow-up to transition these leads into viable diagnostic and therapeutic approaches for managing sepsis and ARDS amidst the COVID-19 pandemic.

## Conclusion

In conclusion, this investigation provides preliminary insights into the possible genetic links between COVID-19, sepsis, and geriatric sepsis-induced ARDS, suggesting potential biomarkers and therapeutic targets for these complex conditions. While the study offers a foundation for exploring innovative immunomodulatory therapies and pharmaceutical compounds, it is important to recognize that these findings are initial and require extensive further research. The targeting of the identified biomarkers and the utilization of suggested drug candidates could represent a promising direction in the efforts to improve patient outcomes for COVID-19 and sepsis/sepsis-ARDS, particularly in the elderly population. However, this must be approached with caution, as further exploration and rigorous validation are necessary to confirm the safety and efficacy of these potential therapeutic interventions. Ultimately, this study contributes to our evolving understanding of the intricate interplay between the immune system, genetics, and the pathogenesis of COVID-19 and sepsis/sepsis-ARDS. It is a stepping stone rather than a definitive solution, and it highlights the need for continued, comprehensive research to pave the way for more effective and validated treatment strategies in the future.

## Data availability statement

The datasets presented in this study can be found in online repositories. The names of the repository/repositories and accession number(s) can be found below: GSE242127 (GEO- https://www.ncbi.nlm.nih.gov/geo/query/acc.cgi?acc=GSE242127).

## Ethics statement

The studies involving humans were approved by Ethics Committees of Zhongshan Hospital. The studies were conducted in accordance with the local legislation and institutional requirements. The participants provided their written informed consent to participate in this study. The animal study was approved by Committee of Animal Experiments at Guangzhou Medical University. The study was conducted in accordance with the local legislation and institutional requirements.

## Author contributions

GQ: Conceptualization, Data curation, Formal Analysis, Investigation, Methodology, Validation, Visualization, Writing – original draft. HWF: Data curation, Formal Analysis, Investigation, Methodology, Validation, Writing – original draft. AC: Data curation, Formal Analysis, Investigation, Methodology, Writing – original draft. ZS: Data curation, Investigation, Methodology, Writing – original draft. MH: Investigation, Methodology, Writing – original draft. ML: Investigation, Methodology, Writing – original draft. EC: Investigation, Methodology, Writing – original draft. SZ: Conceptualization, Funding acquisition, Supervision, Writing – review & editing. XW: Conceptualization, Supervision, Writing – original draft, Writing – review & editing. HF: Conceptualization, Funding acquisition, Supervision, Writing – review & editing.
