# Supplementary material for: A hub gene signature as a therapeutic target and biomarker for sepsis and geriatric sepsis-induced ARDS concomitant with COVID-19 infection
Source: Front Immunol. 2023 Sep 26;14:1257834. doi: 10.3389/fimmu.2023.1257834 (PMC10562607; doi:10.3389/fimmu.2023.1257834)
Supplement: Supplementary file 1 [file DataSheet_1.docx]

**Fig. S1.** The general workflow for this study.


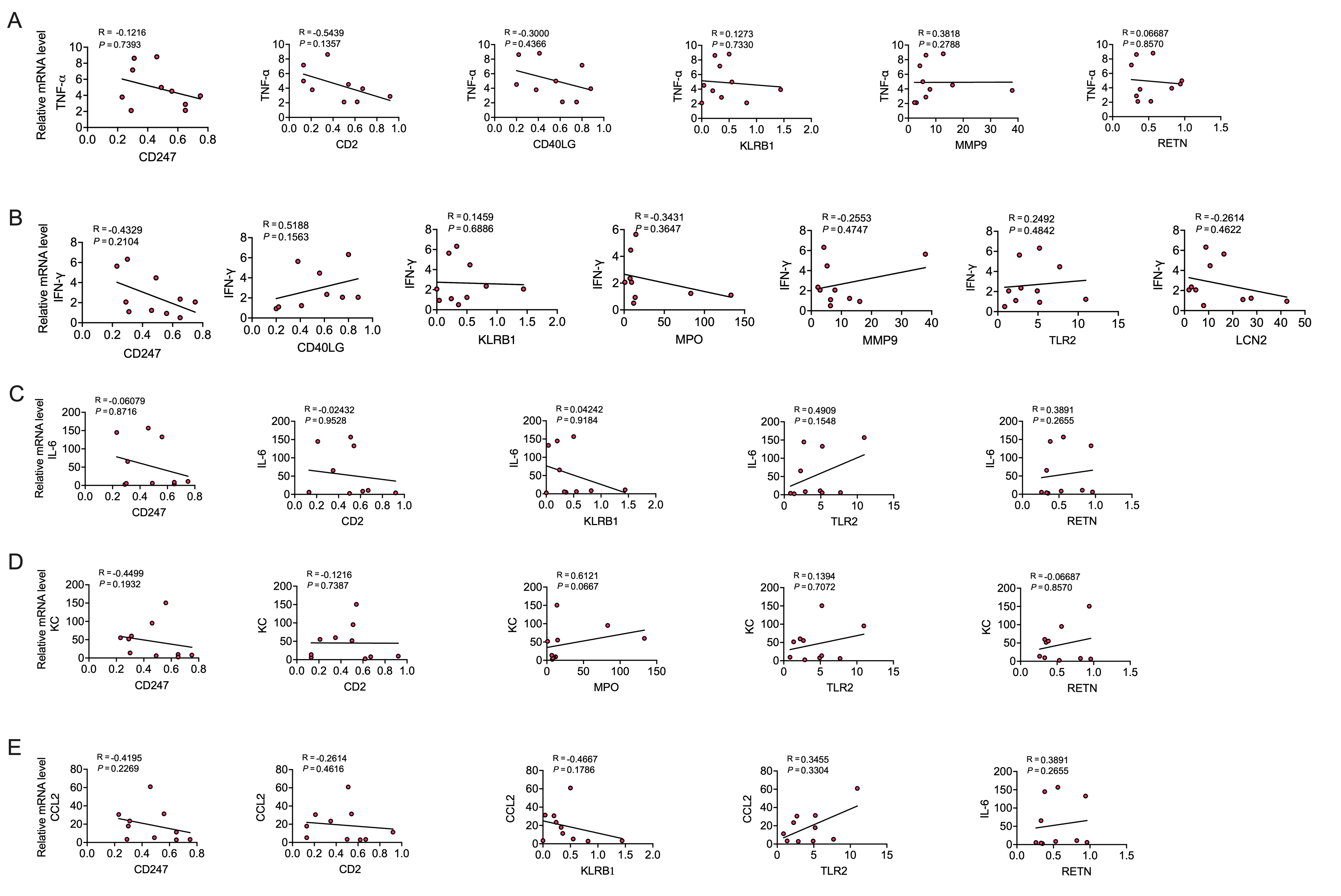


**Fig. S2.** Correlation between identified hub genes and those of TNF-α **(A)**, IFN-γ **(B)**, IL-6 **(C)**, KC **(D)** and CCL-2 **(E)** in murine sepsis-induced ARDS lungs.


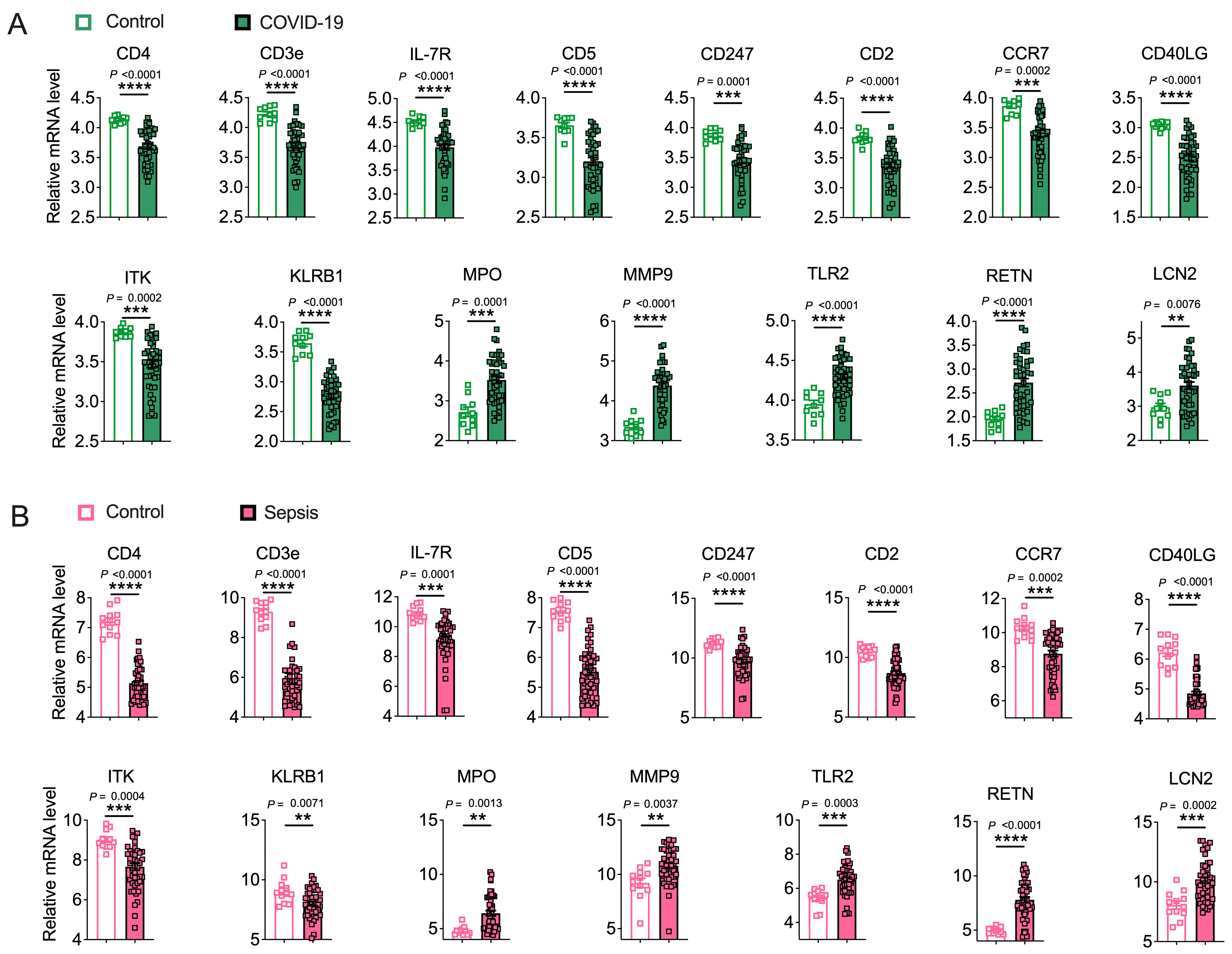


**Fig. S3.** The mRNA expression levels of the identified hub genes were examined in COVID-19 **(A)** and sepsis **(B)**. **(A, B)** Student’s *t*-test.


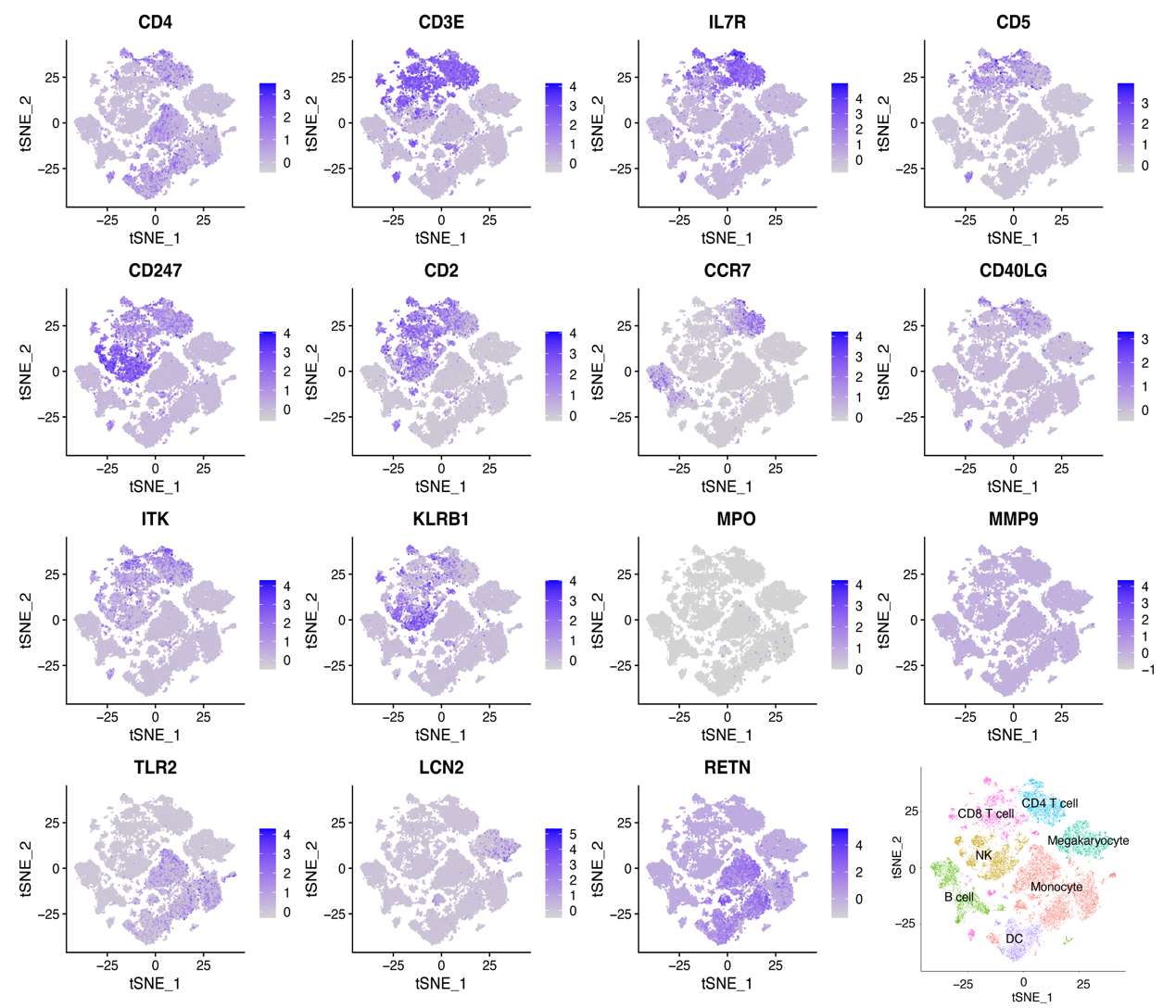


**Fig. S4.** Utilizing scRNA-seq, the identification and characterization of PBMC populations expressing hub genes in elderly ARDS were executed, with t-SNE plots employed for visualization.

**Table S1.** Clinical characteristics of patient cohorts.

| **Cohort:** | **Patients** |
| --- | --- |
| **N** | **17** |
| **Demographics:** |  |
| Age (year) | 72.65±5.92 |
| Sexy (Male/Female) | 14/3 |
| **Underlying medical comorbidities,N(%)** |  |
| Hypertensive disease | 10(58.8) |
| Diabetes mellitus | 6(35.3) |
| Coronary artery disease or congestive heart failure or myocardial infarction | 8(47.1) |
| Chronic lung disease | 4(30.8) |
| Cerebral infarction or Mental disorder | 10(58.8) |
| Chronic kidney disease**^*^** | 0(0) |
| **Clinical information on day of enrollment:** |  |
| Hours from hospital arrival to enrollment | 17.72±10.19 |
| Hours from initial antibiotic initiation | 2.97±1.32 |
| Documented T >=37℃, N(%) | 17(100) |
| Documented SBP <90 mmHg, N(%) | 14(82.4) |
| Vasopressor therapy within 48 hours, N(%) | 8(47.1) |
| **SOFA Score^**^** | 8.59±3.54 |
| **Oxygenation index** | 140.00±51.11 |
| **ARDS classification^***^, (N)** |  |
| *Mild* | 2 |
| *Moderate* | 11 |
| *Severe* | 4 |
| **Clinical lab index:** |  |
| WBC Count (*10^9^/L) | 23.44±10.35 |
| % Neutrophil | 96.95±29.12 |
| % Lymphocytes | 5.34±3.34 |
| % Monocytes | 9.17±20.48 |
| C reactive protein (mg/l) | 176.44±92.91 |
| Procalcitonin, PCT (ng/ml) | 9.95±17.11 |
| Serum lactate (mmol/dL) | 5.37±4.16 |
| **Endotracheal intubation, N(%)** | 13(76.5) |
| **Duration of mechanical ventilation (hours)** | 177.27±159.38 |
| **Clinical outcomes variables:** |  |
| ICU stays (days) | 16.88±9.01 |
| Death during index illness and/or hospitalization,N(%) | 5(41.7) |
| Second hospital admission within 30 days,N(%) | 3(33.3) |
| Mortality(90-day),N(%) | 12(70.6) |

All continuous values were expressed as mean+SD.

^*^ Denotes at least stage 3 chronic kidney disease with glomerular filtration rate <60 mL/min.

^**^ The Sequential organ failure assessment (SOFA) score was calculated at admission to evaluate disease severity based on the functional status of six organ systems.

^***^ The Berlin criteria are employed to diagnose acute respiratory distress syndrome (ARDS) in adult patients.

**Table S2.** Clinical characteristics of three patient cohorts.

All continuous values were expressed as mean+SD.

| **Cohort:** | **Patients** | | | **Analysis** |
| --- | --- | --- | --- | --- |
| **N** | **001** | **002** | **003** | **3** |
| **Demographics:** |  |  |  |  |
| Age (year) | 76 | 68 | 76 | 72.00±5.66 |
| Sexy (Male/Female) | Female | Male | Male | 2/1 |
| **Underlying medical comorbidities, N (%)** |  |  |  |  |
| Hypertensive disease | N | Y | Y | 2(66.7) |
| Diabetes mellitus | Y | N | Y | 2(66.7) |
| Coronary artery disease or congestive heart failure or myocardial infarction | N | N | N | 0 |
| Chronic lung disease | N | N | Y | 1(50) |
| Cerebral infarction or Mental disorder | Y | Y | N | 2(66.7) |
| Chronic kidney disease**^*^** | N | N | N | 0 |
| **Clinical information on day of enrollment:** |  |  |  |  |
| Hours from hospital arrival to enrollment | 10 | 26 | 12 | 16.00±8.72 |
| Hours from initial antibiotic initiation | 3.5 | 1.5 | 3 | 2.67±1.04 |
| Documented T >=37℃, N (%) | Y | Y | Y | 3(100) |
| Documented SBP <90 mmHg, N (%) | Y | Y | Y | 3(100) |
| Vasopressor therapy within 48 hours, N (%) | N | Y | N | 1(50) |
| **Clinical outcomes variables:** |  |  |  |  |
| Duration of mechanical ventilation (hours) | / | 156 | 72 | 114.00±59.40 |
| ICU stays (days) | 16 | 15 | 15 | 15.33±0.58 |
| Death during index illness and/or hospitalization, N (%) | N | N | N | 0 |
| Second hospital admission within 30 days, N (%) | N | N | Y | 1(50) |
| **Clinical parameters** | **D1/D7** | | |  |
| **SOFA Score^**^** | 4/0 | 11/3 | 3/1 |  |
| **Oxygenation index** | 218/336.36 | 127/185.45 | 110/265.41 |  |
| **ARDS classification^***^** | 1/0 | 2/2 | 2/1 |  |
| **Clinical lab index:** |  | | | |
| WBC Count (*10^9^/L) | 26.38/7.53 | 11.02/8.01 | 17.79/6.56 |  |
| % Neutrophil | 88.2/82.6 | 86.7/79.3 | 82.4/72.6 |  |
| % Lymphocytes | 1.7/9.3 | 5.4/9.9 | 8.9/13.6 |  |
| % Monocytes | 8.9/7.0 | 7.7/8.7 | 8.9/13.6 |  |
| C reactive protein (mg/l) | 169.77/36.9 | 136.16/17.53 | 14.85/28.43 |  |
| **Procalcitonin, PCT (ng/ml)** | 19.02/0.15 | 3.58/0.07 | 0.26/0.24 |  |
| **Endotracheal intubation, (Y/N)** | N/N | Y/N | Y/N |  |
| **Body temperature (℃)** | 37.2/36.5 | 36.2/36 | 37.4/36.9 |  |
| **Heart rate (/min)** | 104/65 | 71/68 | 92/76 |  |

^*^ Denotes at least stage 3 chronic kidney disease with glomerular filtration rate <60 mL/min.

^**^ The Sequential organ failure assessment (SOFA) score was calculated at admission to evaluate disease severity based on the functional status of six organ systems.

^***^ Berlin criteria diagnose acute respiratory distress syndrome (ARDS)

**Table S3.** Overview of datasets featuring geo-features and quantitative measurements.

| **Disease name** | **Geo accession** | **GEO platform** | **Total DEGs count** | **Up-regulated DEGs count** | **Down-regulated DEGs count** |
| --- | --- | --- | --- | --- | --- |
| COVID-19 | GSE171110 | GPL16791 | 4082 | 2815 | 1267 |
| Sepsis | GSE137342 | GPL10558 | 551 | 234 | 317 |

**Table S4.** Common genes of COVID-19 and sepsis (*n* = 189).

| ABCA1 | CD96 | FBN2 | KLHL2 | PLEKHB1 | STAT4 |
| --- | --- | --- | --- | --- | --- |
| ADCY4 | CDC20 | FBXO7 | KLHL3 | PLOD1 | STMN3 |
| AGTRAP | CEACAM1 | FCER1A | KLRB1 | POC1A | TBC1D2 |
| ALPL | CEACAM6 | FCER2 | KLRF1 | PRKCH | TCN1 |
| ANKRD22 | CEACAM8 | FCGR1A | KRT72 | PRSS33 | TDRD9 |
| ANXA3 | CHST13 | FCGR1B | LCN2 | PSTPIP2 | TGFBR3 |
| AOC1 | CLEC4D | FCMR | LEF1 | PTGDR2 | TLR2 |
| AP5B1 | CLEC5A | FFAR2 | LILRA3 | PTPN4 | TLR5 |
| ARG1 | COL17A1 | FKBP5 | LILRA5 | PYHIN1 | TMEM204 |
| ATP8B4 | CRIP2 | FLT3LG | LRG1 | RETN | TMEM91 |
| B3GNT8 | CRISPLD2 | FOLR3 | LRPAP1 | RGL4 | TMIGD3 |
| BCAT1 | CST7 | GIMAP5 | LTB4R | RNASE2 | TNFRSF17 |
| BCL11B | CTSG | GINS2 | MAL | RNASE3 | TPI1P2 |
| BMP8B | CTSW | GNLY | MCEMP1 | ROPN1L | TPST1 |
| BPI | CYP1B1 | GOLGA8A | MFSD13A | RPL10A | TRABD2A |
| BST1 | CYSTM1 | GPR183 | MMP9 | RPL13A | TXN |
| C1QB | DDAH2 | GPR84 | MPO | RPL15 | UGCG |
| C3AR1 | DDIAS | GRAMD1A | MS4A3 | RPL22 | UPB1 |
| CA4 | DEFA4 | GYG1 | MYBPC3 | S100A11 | UPP1 |
| CACNA1E | DHRS13 | GYPC | MYL6B | S100A12 | VNN1 |
| CAMP | DRAM1 | HLA-DOA | NELL2 | S100A8 | VPS9D1 |
| CASP5 | DTX3 | HLA-DPA1 | NLRC4 | S100P | VSIG4 |
| CCR3 | EBLN2 | HLA-DRB1 | NRGN | S1PR1 | ZDHHC19 |
| CCR6 | ECHDC3 | HP | OLFM4 | S1PR5 | ZNF683 |
| CCR7 | EEF1A1 | ID3 | OLR1 | SAMD3 |  |
| CD177 | EEF1G | IL18R1 | OPLAH | SAMSN1 |  |
| CD2 | ELANE | IL18RAP | P2RX1 | SCO2 |  |
| CD247 | EMILIN2 | IL32 | PARP10 | SLC26A8 |  |
| CD3E | EOMES | IL7R | PASK | SLC9A1 |  |
| CD4 | EPHX2 | INSL3 | PGLYRP1 | SMARCD3 |  |
| CD40LG | ETS1 | IRAK3 | PHGDH | SORT1 |  |
| CD5 | F5 | ITK | PHTF1 | SPOCK2 |  |
| CD6 | FAM102A | KCNJ2 | PLEKHA1 | SRPK1 |  |

**Table S5.** TF-Gene topology table.

| Id | Label | Degree | Betweenness |
| --- | --- | --- | --- |
| 1236 | CCR7 | 14 | 405.36 |
| 920 | CD4 | 12 | 397.95 |
| 914 | CD2 | 12 | 360.04 |
| 2624 | GATA2 | 11 | 482.83 |
| 2296 | FOXC1 | 9 | 269.21 |
| 959 | CD40LG | 9 | 243.25 |
| 7528 | YY1 | 7 | 221.39 |
| 7097 | TLR2 | 7 | 168.17 |
| 4318 | MMP9 | 7 | 125.76 |
| 921 | CD5 | 7 | 121.2 |
| 3702 | ITK | 6 | 136.65 |
| 3934 | LCN2 | 6 | 136.53 |
| 56729 | RETN | 5 | 147.74 |
| 4782 | NFIC | 5 | 138.02 |
| 916 | CD3E | 5 | 112.9 |
| 3575 | IL7R | 5 | 59.48 |
| 919 | CD247 | 5 | 57.87 |
| 4353 | MPO | 5 | 50.23 |
| 3725 | JUN | 4 | 38.81 |
| 3820 | KLRB1 | 4 | 29.89 |
| 2300 | FOXL1 | 4 | 25.72 |
| 2353 | FOS | 4 | 22.21 |
| 2625 | GATA3 | 3 | 64.02 |
| 860 | RUNX2 | 3 | 49.83 |
| 639 | PRDM1 | 3 | 46.69 |
| 1869 | E2F1 | 3 | 46.54 |
| 5468 | PPARG | 3 | 34.99 |
| 25988 | HINFP | 3 | 16.38 |
| 1051 | CEBPB | 3 | 12.09 |
| 4149 | MAX | 2 | 23.11 |
| 8626 | TP63 | 2 | 16.86 |
| 7392 | USF2 | 2 | 14.58 |
| 5452 | POU2F2 | 2 | 14.52 |
| 6721 | SREBF2 | 2 | 10.39 |
| 3169 | FOXA1 | 2 | 7.68 |
| 6663 | SOX10 | 2 | 5.55 |
| 6722 | SRF | 2 | 5.44 |
| 6720 | SREBF1 | 2 | 4.67 |
| 6774 | STAT3 | 2 | 4.32 |
| 3172 | HNF4A | 2 | 1.11 |
| 6736 | SRY | 1 | 0 |
| 1820 | ARID3A | 1 | 0 |
| 3727 | JUND | 1 | 0 |
| 1385 | CREB1 | 1 | 0 |
| 4205 | MEF2A | 1 | 0 |
| 3202 | HOXA5 | 1 | 0 |
| 4800 | NFYA | 1 | 0 |
| 7020 | TFAP2A | 1 | 0 |
| 4790 | NFKB1 | 1 | 0 |
| 5970 | RELA | 1 | 0 |
| 7157 | TP53 | 1 | 0 |
| 7391 | USF1 | 1 | 0 |
| 6772 | STAT1 | 1 | 0 |
| 7003 | TEAD1 | 1 | 0 |
| 3660 | IRF2 | 1 | 0 |
| 2099 | ESR1 | 1 | 0 |
| 2295 | FOXF2 | 1 | 0 |
| 2908 | NR3C1 | 1 | 0 |
| 5076 | PAX2 | 1 | 0 |
| 4773 | NFATC2 | 1 | 0 |
| 4602 | MYB | 1 | 0 |
| 2001 | ELF5 | 1 | 0 |

**Table S6.** MicroRNA-Gene topology table.

| Id | Label | Degree | Betweenness |
| --- | --- | --- | --- |
| MIMAT0000765 | hsa-mir-335-5p | 3 | 312 |
| MIMAT0026734 | hsa-mir-942-3p | 2 | 1994.31 |
| MIMAT0019041 | hsa-mir-4505 | 2 | 1365 |
| MIMAT0023252 | hsa-mir-5787 | 2 | 1365 |
| MIMAT0018072 | hsa-mir-3652 | 2 | 1356.61 |
| MIMAT0018945 | hsa-mir-4430 | 2 | 1356.61 |
| MIMAT0000449 | hsa-mir-146a-5p | 2 | 1312.13 |
| MIMAT0000435 | hsa-mir-143-3p | 2 | 1242.14 |
| MIMAT0019064 | hsa-mir-4525 | 2 | 545.01 |
| MIMAT0021043 | hsa-mir-5010-5p | 2 | 545.01 |
| MIMAT0027494 | hsa-mir-6797-5p | 2 | 545.01 |
| MIMAT0032029 | hsa-mir-1249-5p | 2 | 545.01 |
| MIMAT0000076 | hsa-mir-21-5p | 2 | 287.17 |
| MIMAT0000062 | hsa-let-7a-5p | 1 | 0 |
| MIMAT0000066 | hsa-let-7e-5p | 1 | 0 |
| MIMAT0000073 | hsa-mir-19a-3p | 1 | 0 |
| MIMAT0000074 | hsa-mir-19b-3p | 1 | 0 |
| MIMAT0000083 | hsa-mir-26b-5p | 1 | 0 |
| MIMAT0000100 | hsa-mir-29b-3p | 1 | 0 |
| MIMAT0000102 | hsa-mir-105-5p | 1 | 0 |
| MIMAT0000231 | hsa-mir-199a-5p | 1 | 0 |
| MIMAT0000255 | hsa-mir-34a-5p | 1 | 0 |
| MIMAT0000256 | hsa-mir-181a-5p | 1 | 0 |
| MIMAT0000265 | hsa-mir-204-5p | 1 | 0 |
| MIMAT0000268 | hsa-mir-211-5p | 1 | 0 |
| MIMAT0000417 | hsa-mir-15b-5p | 1 | 0 |
| MIMAT0000426 | hsa-mir-132-3p | 1 | 0 |
| MIMAT0000441 | hsa-mir-9-5p | 1 | 0 |
| MIMAT0000442 | hsa-mir-9-3p | 1 | 0 |
| MIMAT0000452 | hsa-mir-154-5p | 1 | 0 |
| MIMAT0000458 | hsa-mir-190a-5p | 1 | 0 |
| MIMAT0000510 | hsa-mir-320a | 1 | 0 |
| MIMAT0000680 | hsa-mir-106b-5p | 1 | 0 |
| MIMAT0000683 | hsa-mir-302a-5p | 1 | 0 |
| MIMAT0000730 | hsa-mir-377-3p | 1 | 0 |
| MIMAT0000763 | hsa-mir-338-3p | 1 | 0 |
| MIMAT0000770 | hsa-mir-133b | 1 | 0 |
| MIMAT0000772 | hsa-mir-345-5p | 1 | 0 |
| MIMAT0001631 | hsa-mir-451a | 1 | 0 |
| MIMAT0002174 | hsa-mir-484 | 1 | 0 |
| MIMAT0002175 | hsa-mir-485-5p | 1 | 0 |
| MIMAT0002807 | hsa-mir-491-5p | 1 | 0 |
| MIMAT0002849 | hsa-mir-524-5p | 1 | 0 |
| MIMAT0003294 | hsa-mir-625-5p | 1 | 0 |
| MIMAT0003307 | hsa-mir-637 | 1 | 0 |
| MIMAT0003308 | hsa-mir-638 | 1 | 0 |
| MIMAT0003887 | hsa-mir-769-3p | 1 | 0 |
| MIMAT0004496 | hsa-mir-23a-5p | 1 | 0 |
| MIMAT0004505 | hsa-mir-32-3p | 1 | 0 |
| MIMAT0004513 | hsa-mir-101-5p | 1 | 0 |
| MIMAT0004609 | hsa-mir-149-3p | 1 | 0 |
| MIMAT0004795 | hsa-mir-574-5p | 1 | 0 |
| MIMAT0004910 | hsa-mir-450b-3p | 1 | 0 |
| MIMAT0004917 | hsa-mir-888-3p | 1 | 0 |
| MIMAT0004918 | hsa-mir-892b | 1 | 0 |
| MIMAT0004929 | hsa-mir-190b | 1 | 0 |
| MIMAT0004949 | hsa-mir-877-5p | 1 | 0 |
| MIMAT0004952 | hsa-mir-665 | 1 | 0 |
| MIMAT0005583 | hsa-mir-1228-3p | 1 | 0 |
| MIMAT0005929 | hsa-mir-1275 | 1 | 0 |
| MIMAT0009199 | hsa-mir-365a-5p | 1 | 0 |
| MIMAT0014988 | hsa-mir-3125 | 1 | 0 |
| MIMAT0015025 | hsa-mir-3152-3p | 1 | 0 |
| MIMAT0015029 | hsa-mir-3155a | 1 | 0 |
| MIMAT0015084 | hsa-mir-3199 | 1 | 0 |
| MIMAT0015090 | hsa-mir-1273d | 1 | 0 |
| MIMAT0018086 | hsa-mir-3664-5p | 1 | 0 |
| MIMAT0018097 | hsa-mir-3674 | 1 | 0 |
| MIMAT0018110 | hsa-mir-3682-3p | 1 | 0 |
| MIMAT0018190 | hsa-mir-3916 | 1 | 0 |
| MIMAT0018199 | hsa-mir-3924 | 1 | 0 |
| MIMAT0018931 | hsa-mir-4419a | 1 | 0 |
| MIMAT0018977 | hsa-mir-4455 | 1 | 0 |
| MIMAT0018985 | hsa-mir-3135b | 1 | 0 |
| MIMAT0018997 | hsa-mir-4470 | 1 | 0 |
| MIMAT0019001 | hsa-mir-4474-3p | 1 | 0 |
| MIMAT0019012 | hsa-mir-3155b | 1 | 0 |
| MIMAT0019035 | hsa-mir-4499 | 1 | 0 |
| MIMAT0019047 | hsa-mir-4510 | 1 | 0 |
| MIMAT0019050 | hsa-mir-4513 | 1 | 0 |
| MIMAT0019060 | hsa-mir-4522 | 1 | 0 |
| MIMAT0019739 | hsa-mir-4665-5p | 1 | 0 |
| MIMAT0019788 | hsa-mir-4695-5p | 1 | 0 |
| MIMAT0019821 | hsa-mir-4713-3p | 1 | 0 |
| MIMAT0019838 | hsa-mir-4723-5p | 1 | 0 |
| MIMAT0019849 | hsa-mir-4728-5p | 1 | 0 |
| MIMAT0019853 | hsa-mir-4731-5p | 1 | 0 |
| MIMAT0019855 | hsa-mir-4732-5p | 1 | 0 |
| MIMAT0019921 | hsa-mir-4768-3p | 1 | 0 |
| MIMAT0019926 | hsa-mir-4772-5p | 1 | 0 |
| MIMAT0019931 | hsa-mir-4775 | 1 | 0 |
| MIMAT0019938 | hsa-mir-4779 | 1 | 0 |
| MIMAT0019942 | hsa-mir-4781-5p | 1 | 0 |
| MIMAT0019955 | hsa-mir-4786-3p | 1 | 0 |
| MIMAT0021130 | hsa-mir-5197-5p | 1 | 0 |
| MIMAT0022469 | hsa-mir-5681a | 1 | 0 |
| MIMAT0022491 | hsa-mir-5698 | 1 | 0 |
| MIMAT0022717 | hsa-mir-873-3p | 1 | 0 |
| MIMAT0022833 | hsa-mir-365b-5p | 1 | 0 |
| MIMAT0023702 | hsa-mir-6077 | 1 | 0 |
| MIMAT0023715 | hsa-mir-6090 | 1 | 0 |
| MIMAT0024610 | hsa-mir-6127 | 1 | 0 |
| MIMAT0024613 | hsa-mir-6129 | 1 | 0 |
| MIMAT0024614 | hsa-mir-6130 | 1 | 0 |
| MIMAT0024615 | hsa-mir-6131 | 1 | 0 |
| MIMAT0024617 | hsa-mir-6133 | 1 | 0 |
| MIMAT0026478 | hsa-mir-133a-5p | 1 | 0 |
| MIMAT0026719 | hsa-mir-889-5p | 1 | 0 |
| MIMAT0027468 | hsa-mir-6784-5p | 1 | 0 |
| MIMAT0027470 | hsa-mir-6785-5p | 1 | 0 |
| MIMAT0027550 | hsa-mir-6825-5p | 1 | 0 |
| MIMAT0027564 | hsa-mir-6832-5p | 1 | 0 |
| MIMAT0027583 | hsa-mir-6840-3p | 1 | 0 |
| MIMAT0027587 | hsa-mir-6842-3p | 1 | 0 |
| MIMAT0027611 | hsa-mir-6855-3p | 1 | 0 |
| MIMAT0027618 | hsa-mir-6859-5p | 1 | 0 |
| MIMAT0027634 | hsa-mir-6867-5p | 1 | 0 |
| MIMAT0027640 | hsa-mir-6870-5p | 1 | 0 |
| MIMAT0027666 | hsa-mir-6883-5p | 1 | 0 |
| MIMAT0027668 | hsa-mir-6884-5p | 1 | 0 |
| MIMAT0028119 | hsa-mir-7111-5p | 1 | 0 |
| MIMAT0030412 | hsa-mir-7843-3p | 1 | 0 |
| MIMAT0030979 | hsa-mir-8052 | 1 | 0 |
| MIMAT0031890 | hsa-mir-203a-5p | 1 | 0 |
| MIMAT0035542 | hsa-mir-9500 | 1 | 0 |
